# An Unusual Presentation of Stroke and Reperfusion: A Case Report

**DOI:** 10.7759/cureus.33360

**Published:** 2023-01-04

**Authors:** Ira S Nash, Beth Nash, Richard Libman

**Affiliations:** 1 Department of Cardiology, The Donald and Barbara Zucker School of Medicine at Hofstra/Northwell, Hempstead, USA; 2 Internal Medicine, Consultant, Scarsdale, USA; 3 Department of Neurology, The Donald and Barbara Zucker School of Medicine at Hofstra/Northwell, Hempstead, USA

**Keywords:** reperfusion, tissue plasminogen activator (tpa), déjà vu, case report, left ventricular apical aneurysm, revascularization of emboblic stroke, embolic stroke, stroke

## Abstract

Intravenous tissue plasminogen activator (tPA) is a mainstay of therapy in acute ischemic stroke but transient neurologic changes related to reperfusion have not been well described. One of the authors (ISN) experienced a cardioembolic stroke due to apical hypertrophic cardiomyopathy with a left ventricular apical aneurysm. He received tPA and we describe his unusual cognitive symptoms during the infusion. The patient’s presenting neurologic deficit improved with tPA, suggesting reperfusion. His subsequent restlessness, disorientation, and déjà vu lasted about 10 minutes and resolved spontaneously. Imaging studies confirmed an ischemic infarction in the left posterior cerebral artery (PCA) distribution. Cardiac events, including arrhythmias related to coronary reperfusion after myocardial infarction, are well described. Neurologic events due to reperfusion have not been previously described in patients with stroke. We describe a case of transient neurologic symptoms during revascularization of an embolic stroke.

## Introduction

Ventricular cardiac arrhythmias are a well-described phenomenon accompanying the restoration of coronary artery patency in the setting of acute myocardial infarction. In the early experience of using thrombolytic therapy for acute myocardial infarction, successfully achieving patency of the occluded coronary artery was so frequently accompanied by “reperfusion arrhythmias” that a transient accelerated idioventricular rhythm was commonly accepted as a non-invasive sign of therapeutic success [1]. An analogous phenomenon of abnormal brain activity following restoration of cerebral blood flow in an acute ischemic stroke has not previously been described. We report a case that suggests the occurrence of a brain reperfusion syndrome in the setting of restoration of cerebral vessel patency following an acute cardioembolic stroke.

## Case presentation

A 63-year-old man (ISN) experienced sudden onset of tingling in his right occipital region and right homonymous hemianopia, along with mild paresthesia of the right arm. He had a history of apical hypertrophic cardiomyopathy with apical aneurysm and preserved left ventricular systolic function. A subcutaneous implantable cardioverter defibrillator (Emblem MRI S-ICD, Boston Scientific) had been placed 16 months earlier, following several episodes of non-sustained ventricular tachycardia. He was asymptomatic with a normal exercise tolerance and had not experienced any implantable cardioverter-defibrillator (ICD) discharges. He had no history of hypertension, diabetes, smoking or atrial fibrillation, and no episodes of atrial fibrillation had been detected by his ICD. His only medications were alfuzosin 10 mg daily, atorvastatin 20 mg daily and metoprolol succinate 25 mg daily.

**Figure 1:**
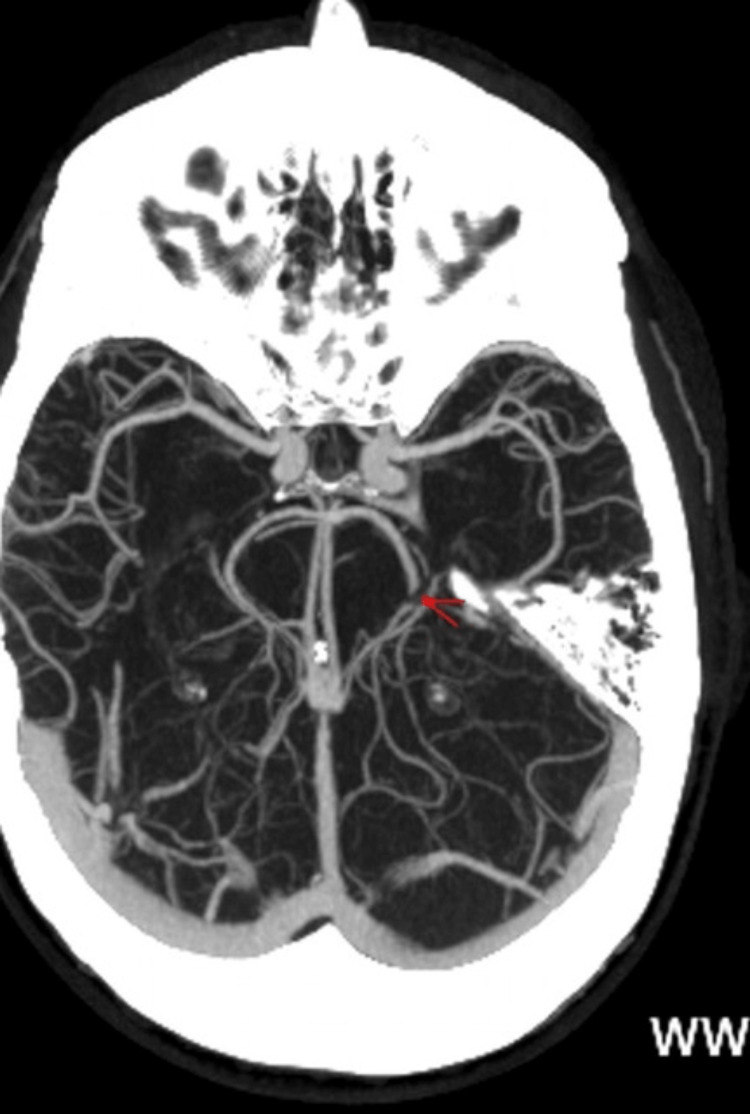
CT angiogram demonstrating a filling defect with abrupt cut-off of flow in the left PCA P3 segment with reconstitution of flow distally PCA: posterior cerebral artery

The patient promptly recognized that he was having a stroke and activated emergency medical services. At the time of hospital transport, the tingling in his right occipital region had progressed to mild right posterior headache, and his visual field deficit had improved to a right superior quadrantanopia. His blood pressure was 106/65 mmHg with a heart rate of 60 bpm and arterial oxygen saturation of 98% on room air. He remained awake, alert, and oriented to person, place and time, though mildly confused, or amnestic on deeper questioning. Neurologic examination showed a right superior quadrantanopia and a mild right upper extremity pronator drift and was otherwise normal. Urgent CT head/CT angiography of the neck and head demonstrated a filling defect with an abrupt cut-off of flow in the left posterior cerebral artery (PCA) P3 segment, approximately 5 mm in length with reconstitution of flow distally. The common carotid, internal carotid, and vertebral arteries were patent with no hemodynamically significant stenosis. There was no evidence of intracranial hemorrhage (Figure 1). Based on the angiographic findings, clinical presentation and the patient's low risk for bleeding, intravenous tissue plasminogen activator (tPA) was administered approximately 90 minutes after symptom onset.

Approximately 30-40 minutes after the start of the tPA infusion, the patient became suddenly agitated and restless. He reported feeling generally unwell, with an intense sense of déjà vu, insisting (contrary to fact) that he had been in the same emergency department weeks earlier and was struggling to remember the reason. An observer (BN) reported that he had a blank stare and struggled to describe what he was experiencing. His vital signs and prior neurologic deficits were stable, and he remained alert and responsive, but was aware of (and disturbed by) his symptoms, which resolved spontaneously within about 10 minutes. Shortly after completion of the tPA infusion he reported that his visual field deficit had resolved, which was supported by confrontation visual field testing.

**Figure 2:**
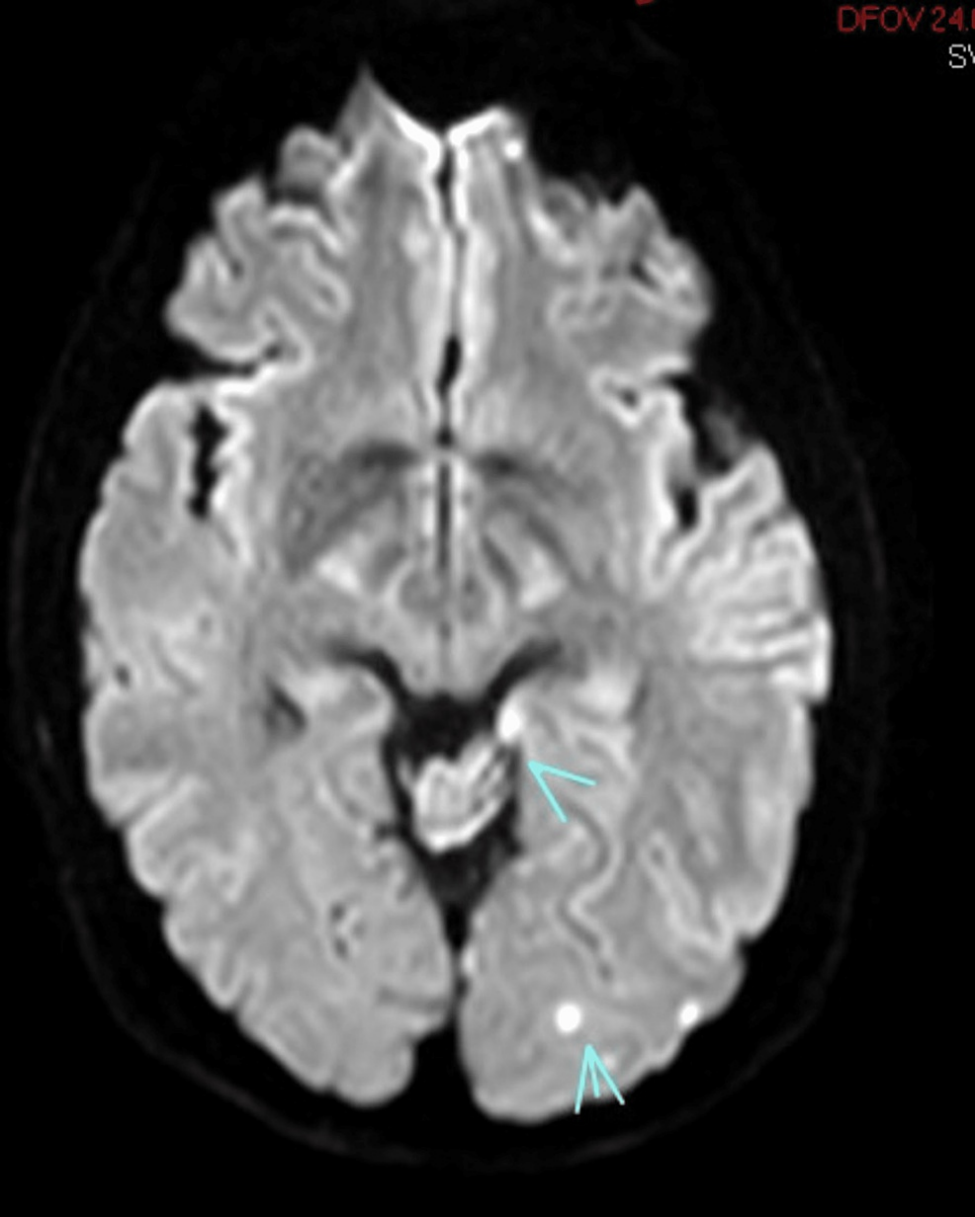
MRI of the brain demonstrating foci of acute/early subacute ischemic infarction in the left PCA distribution, involving the left occipital cortex and the posterior body to tail of the hippocampus PCA: posterior cerebral artery

He was admitted to the intensive care unit and experienced no further neurologic signs or symptoms. Headache and right arm symptoms resolved over the next several hours. Blood pressure and heart rate remained normal. An MRI of the brain, performed the next day, showed a few small foci of acute/early subacute ischemic infarction in the left PCA distribution, involving the left occipital cortex and the posterior body to tail of the hippocampus (Figure 2). A technically limited bedside echocardiogram failed to identify an intracardiac thrombus. An EEG was not performed given the transience of his déjà vu and the absence of any recurrence. The patient was discharged after a two-night stay with complete resolution of his neurologic deficits and with instructions to start rivaroxaban 20 mg daily. An echocardiogram three days after discharge confirmed the prior finding of a left ventricular apical aneurysm, but no intracardiac thrombus was visible and no valvular abnormalities were seen. A bubble study was normal.

## Discussion

Although there was no cardiac thrombus detected, the diagnosis of a cardioembolic stroke of left ventricular origin was based on the known association of the anatomic finding with thromboembolism, the absence of significant atherosclerotic disease in the aortic arch or cervicocephalic vessels, and the absence of any evidence of atrial fibrillation or paradoxical embolization [2]. The patient had a highly favorable response to thrombolytic therapy, likely related in part to the rapid initiation of treatment [3].

The transient restlessness, disorientation, and déjà vu during tPA infusion in this patient may represent a previously unreported cerebral reperfusion reaction, similar to what has been described during reperfusion of ischemic injury to the heart. Reperfusion of ischemic myocardial tissue following vascular occlusion can lead to a cascade of adverse reactions, provoked by reactive hyperemia and generation of abnormal levels of free radicals and other reactive species [4-5]. In reperfused myocardium in the setting of acute myocardial infarction, reperfusion ventricular arrhythmias and contractile dysfunction are commonplace.

We believe the constellation of symptoms reported here resulted from successful thrombolysis and represent a previously unreported analogous brain reperfusion syndrome. While we cannot rule out a focal unaware seizure provoked by PCA distribution infarction, or behavioral changes associated with PCA infarction as part of the “top of the basilar” syndrome, our conclusion is based on: the timing of the symptoms during the infusion of thrombolytic agent; the simultaneous resolution of the visual field deficit with the onset of the déjà vu; the biologic plausibility of enhanced brain synaptic activity in the previously ischemic vascular distribution, including the visual cortex and the meso-temporal lobe, which is thought to be where déjà vu localizes; and the transient nature of the symptoms [6].

## Conclusions

We present a case of successful thrombolysis of cardioembolic stroke complicated by transient agitation and intense déjà vu that may represent a previously unreported or poorly recognized syndrome of brain reperfusion. We propose that the pathophysiology of this syndrome is similar to the abnormal cellular behavior seen in myocardial tissue following successful restoration of blood flow in ischemic injury. Further study is needed to determine how often abnormal brain activity occurs in the setting of restoration of cerebral vessel patency, and if it presents in different ways depending on the location of the ischemic injury. Clarification of the frequency and pattern of this phenomenon could provide physicians with a non-invasive marker of the success of cerebral revascularization in acute ischemic stroke.

## References

[1] Gorgels APM, Vos MA, Letsch IS (1988). Usefulness of the accelerated idioventricular rhythm as a marker for myocardial necrosis and reperfusion during thrombolytic therapy in acute myocardial infarction. Am J Cardiol.

[2] Rowin EJ, Maron BJ, Haas TS, Garberich RF, Wang W, Link MS, Maron MS (2017). Hypertrophic cardiomyopathy with left ventricular apical aneurysm: implications for risk stratification and management. J Am Coll Cardiol.

[3] Emberson J, Lees KR, Lyden P (2014). Effect of treatment delay, age, and stroke severity on the effects of intravenous thrombolysis with alteplase for acute ischaemic stroke: a meta-analysis of individual patient data from randomised trials. Lancet.

[4] Okamura A, Ito H, Iwakura K (2006). Effect of reactive hyperemia after coronary recanalization on myocardial tissue reperfusion by thrombolysis in myocardial infarction flow grade in acute myocardial infarction. Am J Cardiol.

[5] Cannon RO 3rd (2005). Mechanisms, management and future directions for reperfusion injury after acute myocardial infarction. Nat Clin Pract Cardiovasc Med.

[6] Caplan LR (1980). "Top of the basilar" syndrome. Neurology.

